# Human Bocavirus Infection, Canada

**DOI:** 10.3201/eid1205.051424

**Published:** 2006-05

**Authors:** Nathalie Bastien, Ken Brandt, Kerry Dust, Diane Ward, Yan Li

**Affiliations:** * National Science Center for Human and Animal Health, Winnipeg, Manitoba, Canada;; † Saskatchewan Health, Regina, Saskatchewan, Canada

**Keywords:** Parvoviruses, Bocavirus, HBoV, respiratory infection, dispatch

## Abstract

Human Bocavirus was detected in 18 (1.5%) of 1,209 respiratory specimens collected in 2003 and 2004 in Canada. The main symptoms of affected patients were cough (78%), fever (67%), and sore throat (44%). Nine patients were hospitalized; of these, 8 (89%) were <5 years of age.

A new parvovirus, human Bocavirus (HBoV), was recently identified in Sweden (*1*). The virus was identified in clinical specimens from infants and children with respiratory tract illness. Phylogenetic analyses of the complete genome of HBoV showed that that the virus is most closely related to canine minute virus and bovine parvovirus, which are members of the genus *Bocavirus*, family *Parvoviridae* (*1*). To date, the only parvovirus known to be pathogenic in humans is B19, which is responsible for Fifth disease in children (*2*). The role of HBoV in respiratory tract illnesses is unknown. We retrospectively investigated HBoV in Canadian patients with acute respiratory infection (ARI) in 2003 and 2004 to assess the impact of HBoV infections on respiratory tract illnesses and identify the signs and symptoms of this illness.

## The Study

A total of 1,209 specimens from patients with ARI from January 2003 to December 2004 were tested for HBoV. The specimens originated from the Saskatchewan provincial public health laboratories. Specimen types analyzed included throat swabs, nasopharyngeal swabs, nasopharyngeal aspirates, and auger suctions. All specimens were negative for influenza viruses A and B; parainfluenza viruses 1, 2, and 3; adenovirus; and respiratory syncytial virus (RSV) by direct or indirect fluorescence assays or virus isolation and for human metapneumovirus (HMPV) by reverse transcription–polymerase chain reaction. Specimens were collected from all age groups: 290 (24%) from those <5 years of age, 59 (5%) from those 6–10 years of age, 90 (7.4%) from those 11–15 years of age, 86 (7.1%) from those 16–20 years of age, 358 (29.6%) from those 21–50 years of age, and 324 (27%) from those >50 years of age, The age of the patients was unknown for 2 (0.2%) specimens.

HBoV was detected by polymerase chain reaction (PCR) using primers specific for 2 different regions of the genome. The screening primers 188F (2281-5´-GACCTCTGTAAGTACTATTAC-3´-2301) and 542R (2634-5´-CTCTGTGTTGACTGAATACAG-3´-2614), reported by Allander et al. (*1*), were based on the sequence of the putative noncapsid protein 1 (NP-1) gene. The second set of primers, VP1/VP2F (4492-5´-GCAAACCCATCACTCTCAATGC-3´-4513) and VP1/VP2R (4895-5´-GCTCTCTCCTCCCAGTGACAT-3´-4875), was used for confirmation and was based on the published HBoV putative VP1/VP2 gene sequences (DQ000495) (*1*). Viral DNA was extracted from 285 μL of original samples with a BioRobot MDx and the QiAamp Virus BioRobot MDX kit (Qiagen, Valencia, CA, USA). We used 5 μL of DNA in a volume of 50 μL containing 20 pmol of each primer. The thermocycler conditions were 95°C for 15 min for activation of HotStartTaq DNA polymerase (Qiagen); 35 cycles of 94°C for 1 min, 54°C for 1 min, and 72°C for 2 min; and extension at 72°C for 10 min. Nucleotide sequences of NP-1 gene amplicons were determined with an ABI 377 Sequencer and a fluorescent dye terminator kit (Applied Biosystems, Foster City, CA, USA). DNA sequences were assembled and analyzed with SEQMAN, EDITSEQ, and MEGALIGN programs in Lasergene (DNASTAR, Madison, WI, USA). To avoid cross-contamination, specimen processing, DNA extraction, amplification, and analyses were conducted in different rooms. For DNA extraction and PCR procedures, we included 12 negative controls per 96-well plate.

A total of 18 (1.5%) of the 1,209 specimens tested were positive for HBoV by PCR. HBoV activity was found throughout the year with no apparent seasonal prevalence (Table 1). The sex distribution of patients was 61% (11) male and 39% (7) female (Table 2). Patients with HBoV ranged in age from 10 months to 60 years (median 11.5 years), and no significant difference in infection rates was observed between age groups.

**Table 1:** Distribution of human Bocavirus–positive specimens by month, Canada

Date	No. positive/no. tested
2003	
Jan	1/48
Feb	2/51
Mar	0/44
Apr	1/49
May	0/48
Jun	4/54
Jul	1/53
Aug	0/46
Sep	0/49
Oct	0/51
Nov	1/50
Dec	1/50
2004	
Jan	0/50
Feb	1/51
Mar	0/48
Apr	0/50
May	0/50
Jun	0/50
Jul	1/50
Aug	0/66
Sep	1/52
Oct	0/49
Nov	3/50
Dec	1/50

**Table 2:** Data from medical files of patients infected with human Bocavirus, Canada*

Specimen no.	Date collected	Sex	Patient status	Age	Symptoms
883	Jan 17, 2003	M	O	23 y	Fever, cough
947	Feb 5, 2003	M	H	9 mo	Fever, cough, nausea, rhinitis, pneumonia
963	Feb 28, 2003	F	O	11 y	Fever, cough, sore throat, rhinitis
1029	Apr 10, 2003	F	O	16 y	Sore throat, headache
1122	Jul 8, 2003	F	H	1 y	Fever, cough
1166	Jun 10, 2003	M	O	17 y	Sore throat, rhinitis
1178	Jun 16, 2003	M	H	28 y	Fever
1179	Jun 16, 2003	F	H	3 y	Fever, cough, rhinitis
1181	Jun 18, 2003	M	H	1 y	Fever, cough, rhinitis
1368	Nov 1, 2003	F	O	60 y	Cough, flulike symptoms, myalgia, headache, nausea
1431	Dec 16, 2003	F	O	41 y	Cough, sore throat, flulike symptoms, bronchiolitis
1545	Feb 24, 2004	M	H	10 mo	Fever, cough, pneumonia
1776	Jul 12, 2004	M	H	2 y	Fever
1871	Sep 3, 2004	M	H	11 mo	Cough, rhinitis, bronchiolitis, pneumonia
1979	Nov 8, 2004	F	O	12 y	Fever, cough, sore throat, flulike symptoms, headache
2013	Nov 28, 2004	M	H	9 mo	Cough
2016	Nov 25, 2004	F	O	14 y	Fever, cough, sore throat, flulike symptoms
2021	Dec 1, 2004	M	O	37 y	Fever, cough, sore throat, flulike symptoms, headache

*O, outpatient; H, hospitalized.

The main clinical symptoms were cough (78%), fever (67%), and sore throat (44%) (Table 2). Other clinical symptoms included flulike symptoms (28%), headache (22%), nausea (17%), and myalgia (11%). Five patients had rhinitis, 1 had pneumonia, and 1 had bronchiolitis. One patient had rhinitis, bronchiolitis, and pneumonia, and 1 patient had rhinitis and pneumonia. Nine (50%) HBoV patients were hospitalized; 8 (89%) were <5 years of age, and 1 was between 21 and 50 years of age. The incidence of lower respiratory tract infection was lower in outpatients: 1 with bronchiolitis and no pneumonia (Table 2). Although the infection rates were similar in all age groups, a significant increase in hospitalization rates was seen in those <5 years of age compared with those >6 years of age (8/8 vs. 1/10, p = 0.001) (Table 2). All patients with pneumonia (3/3) and half of those with bronchiolitis (1/2) were also in this age group.

Nucleotide sequences were determined for nucleotides 2342–2581 that encode the NP-1 gene of HBoV (GenBank accession nos. DQ267760–DQ267775). No differences in nucleic acid sequences were found between different Canadian isolates. These isolates were also identical to 2 Swedish isolates (ST1 and ST2, GenBank accession nos. DQ000495–DQ000496) (*1*).

## Conclusions

Although a causal relationship still needs to be demonstrated by including a control group of healthy persons, detection of HBoV in respiratory tract specimens from patients with undiagnosed ARI suggests that this virus may be associated with respiratory illness. This finding supports those of Allander et al. with regard to the association of HBoV with respiratory disease (*1*). It also demonstrates that HBoV was present in Canada in 2003 and 2004, which suggests that it may be circulating worldwide. Since this study used only samples from ARI patients who tested negative for influenza viruses A and B, parainfluenza viruses 1–3, adenovirus, RSV, and HMPV, dual infection cannot be excluded. In addition, whether HBoV is present asymptomatically in humans cannot be excluded because samples from healthy persons were not tested.

Allander et al. reported HBoV only in infants and children, which was probably the result of testing fewer specimens from adults patients (*1*). Most respiratory viruses show a seasonal distribution with peak activity in winter. Human parvovirus B19, the only parvovirus that is pathogenic in humans, is also seasonal, with peak occurrences in spring and summer (*3*). In contrast, no seasonal prevalence was observed for HBoV infection; the virus was found throughout the year. The lack of seasonality observed for HBoV may have been caused by the low prevalence in this study. Thus, additional year-round studies are needed to better understand the epidemiology of HBoV. Most (89%) hospitalizations were in persons <5 years of age, which suggests that HBoV may cause more severe respiratory illness in infants and children, similar to disease caused by RSV (*4**,**5*), HMPV (*6**,**7*), human coronavirus NL63 (*8**–**14*), and human coronavirus 229E (*15*). More comprehensive studies with data on prevalence, risk factors, and use of health services are needed to determine the role of HBoV in ARI and its effect on the healthcare system.
